# Association between long-term neuro-toxicities in testicular cancer survivors and polymorphisms in glutathione-s-transferase-P1 and -M1, a retrospective cross sectional study

**DOI:** 10.1186/1479-5876-5-70

**Published:** 2007-12-27

**Authors:** Jan Oldenburg, Sigrid M Kraggerud, Marianne Brydøy, Milada Cvancarova, Ragnhild A Lothe, Sophie D Fossa

**Affiliations:** 1 Department of Clinical Cancer Research, The Norwegian Radiumhospital, Oslo, Norway; 2 Faculty of medicine, University of Oslo, 0310 Oslo, Norway; 3 Department of Cancer Prevention, Institute for Cancer Research, The Norwegian Radiumhospital, and University of Oslo, Norway; 4 Centre for Cancer Biomedicine, University of Oslo, Norway; 5 Department of Oncology and Medical Physics, Haukeland University Hospital, Bergen, Norway; 6 Section of Oncology, Institute of Medicine, University of Bergen, Bergen, Norway; 7 Section of Biostatistics, The Norwegian Radiumhospital, Oslo, Norway

## Abstract

**Background:**

To assess the impact of polymorphisms in Glutathione S-transferase (GST) -P1, -M1, and -T1 on self-reported chemotherapy-induced long-term toxicities in testicular cancer survivors (TCSs).

**Methods:**

A total of 238 TCSs, who had received cisplatin-based chemotherapy at median twelve years earlier, had participated in a long-term follow-up survey which assessed the prevalence of self-reported paresthesias in fingers/toes, Raynaud-like phenomena in fingers/toes, tinnitus, and hearing impairment. From all TCSs lymphocyte-derived DNA was analyzed for the functional A→G polymorphism at bp 304 in *GSTP1*, and deletions in *GST-M1 *and *GST-T1*. Evaluation of associations between GST polymorphisms and self-reported toxicities included adjustment for prior treatment.

**Results:**

All six evaluated toxicities were significantly associated with the cumulative dose of cisplatin and/or bleomycin. Compared to TCSs with either *GSTP1-AG *or *GSTP1*-*AA*, the 37 TCSs with the genotype *GSTP1-GG*, were significantly less bothered by paresthesias in fingers and toes (p = 0.039, OR 0.46 [0.22–0.96] and p = 0.023, OR 0.42 [0.20–0.88], respectively), and tinnitus (p = 0.008, OR 0.33 [0.14–0.74]). Furthermore, absence of functional GSTM1 protected against hearing impairment (p = 0.025, OR 1.81 [1.08–3.03]).

**Conclusion:**

In TCSs long-term self-reported chemotherapy-induced toxicities are associated with functional polymorphisms in *GSTP1 *and *GSTM1*. Hypothetically, absence of GST-M1 leaves more glutathione as substrate for the co-expressed GST-P1. Also intracellular inactivation of pro-apoptotic mediators represents a possible explanation of our findings. Genotyping of these GSTs might be a welcomed step towards a more individualized treatment of patients with metastatic testicular cancer.

## Background

The progress of cisplatin-based chemotherapy has changed the once dark prospects of disseminated testicular cancer (TC) into a model of a curable neoplasm [1,2]. This success story of oncological treatment however, is shadowed by an increase in reports on long-term somatic and psychosocial sequalae in testicular cancer survivors (TCSs) [3,4]. Cisplatin (C) is the cornerstone of chemotherapeutic treatment of TC. It has usually been combined with bleomycin (B), and vinblastine (V) as CVB regimen, until Williams et al. 1987 demonstrated that substitution of vinblastine by etoposide (E) as BEP regime resulted in increased survival and less toxicities [5]. While peripheral neurotoxicity can be induced by several of these drugs [6-8], ototoxicity (tinnitus and/or impaired hearing) is mainly ascribed to cisplatin [9,10]. Clinicians have observed large inter-individual variations of treatment-induced toxicities among patients after similar chemotherapy regimens. In animal models these variations have been greater than explainable by variable pharmacokinetic properties [11]. These observations led to the assumption of genetic differences in the elimination, detoxification and/or toleration of cytotoxic agents among patients.

Glutathione S-Transferases (GSTs) are enzymes which have been linked to both the etiology of testicular cancer [12], the cure rate of platinum-based chemotherapy [13], and chemotherapy-induced toxicities [14]. GSTs are expressed in the testicles and levels of GTP1 are increased in case of acquired resistance to cisplatin [15,16]. GSTs might moderate toxicities systemically but expression of these enzymes at the affected site increases the credibility of potential associations. GST-M1 and GST-P1 are both expressed at the corti organ,[17] which is affected in case of cisplatin-induced ototoxicity. Both enzymes are also expressed in dorsal root ganglion cells,[18] which are known to be damaged by cisplatin [19]. Further functions of GSTs have been reviewed in detail elsewhere [20,21]. We considered germ-line polymorphisms within GST genes as promising candidates for the exploration of the large inter-individual variability of long-term treatment toxicities. Recently, we reported on their importance for cisplatin-induced audiometric assessed hearing impairment [22]. In the present study we aimed to investigate the relevance of functional genotype polymorphisms of *GST-M1,-P1 and-T1 *for the prevalence of self-reported long-term paresthesias, Raynaud-like phenomena, tinnitus, and hearing impairment in a large sample of cisplatin-based chemotherapy treated TCSs.

## Methods and patients

### Patients

During the years 1998 to 2002, all Norwegian TCSs treated between 1980 and 1994 and aged between 18 and 75 years were invited to participate in a long-term survey, which consisted of a questionnaire and an out-patient visit. Patients with evidence of active TC, extragonadal germ cell malignancy, bilateral TC, a second non-germ cell malignancy (except skin cancer), and those, in whom the non-affected testicle had been removed previously due to a benign condition, were excluded [23]. Post-orchiectomy treatment was principally applied according to specified protocols as described previously [24]. Most patients treated by chemotherapy received cisplatin (C) in combination with bleomycin (B), etoposide (E) or vinblastine (V) as 3–4 courses of BEP or CVB. These regimens were applied in conjunction with a rigorous hydration regime in all patients. For the purpose of the present study, the type of regimen, cumulative dosage per square meter body surface [mg/m^2^] of the most commonly used cytotoxic substances, exact number of cycles and dates of application were retrieved from treatment charts. The present study includes only TCSs, who have been treated with cisplatin-based chemotherapy at the Norwegian Radium Hospital (NRH) and for whom lymphocytes have been available for DNA analyses.

### Genotyping

Whole blood EDTA samples were collected from the TCSs, lymphocyte-DNA extracted, and submitted to genetic analyses of functional polymorphisms in the genes coding for *GSTT1*, -*M1*, and -*P1*. 173 of the 238 TCSs included in this study are the same as those previously reported by us [22]. The known inherited homozygous deletions in *GSTT1 *and *GSTM1 *are equivalent to non-functional enzymes. A functional single nucleotide polymorphism (SNP) in the *GSTP1 *gene at base pair 315 between Adenosine (A) and Guanine (G) leads to the expression of either isoleucine (Ile) or valine (Val) at codon 105 (Ile105, Val105).

GST analysis was performed according to a previously described multiplex PCR protocol [25]. Briefly, 50–100 ng DNA and 30 pmol of each of the primers for *GSTM1*, *GSTT1*, and *GSTP1*, and 10 pmol of the GSTmu2 antisense primer, 1.4 mM MgCl_2_, PCR buffer II from Perkin Elmer (MgCl_2 _free), and 0.75 Units Taq polymerase were mixed. The fragment lengths of the PCR products were 480 base pairs (bp) for *GSTT1*, 294 for *GSTP1*, 275 for *GSTM1*, and 175 bp for *GSTM2 *which served as a positive PCR control. Subsequently, 20 μl of the PCR product (unpurified) was digested with 8 Units of the restriction enzyme Alw261 (MBI Fermentas, USA), and the fragments were separated by agarose (4%) gel (NuSieve 3:1, FCM Bioproducts, Rockland, ME, USA) electrophoresis. The PCR products of *GSTT1 *and *GSTM2 *remain uncut, whereas the *GSTP1 *fragment is cut into 234 bp and 60 bp if codon 105 contains a G (guanine). *GSTM1 *contains a nonpolymorphic Alw1261 restriction site and therefore the PCR product is digested to 195 and 80 bp long fragments in all samples positive for *GSTM1 *alleles, (*GSTM1****+***).

### Questionnaire module (SCIN)

**Figure 1:**
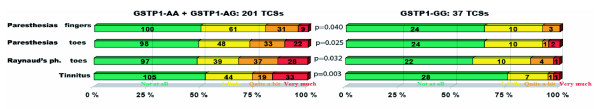
Prevalence paresthesias in the fingers and toes, Raynaud-like phenomena in the toes, and tinnitus in TCSs depending on the GSTP1-GG genotype, statistical testing by χ^2^-trends.

In this study we use a TC specific quality of life (QLQ) module which was designed by Fossa et al. and used together with the EORTC QLQ-C30 [26]. This module was based on interviews with TCSs and reviews of the available literature on long-term morbidity in TCSs and was subsequently slightly modified and psychometrically evaluated. It covers six symptoms (items), figure 1, and is named: Scale for Chemotherapy-Induced Neurotoxicity (SCIN) [27]. Summation of all six item scores, ranging from 0 to 3, yields the SCIN-total-score which we consider representing the overall chemotherapy-induced neurotoxicity. In the present study we divided the SCIN-total-score into three symptom-classes: none (0–5), moderate (6–9), and severe (10–18).

The SCIN's psychometric properties have been validated and were considered suitable for its application as screening instrument for chemotherapy-induced neurotoxicity [27].

### Statistical analyses

The above described item scores and the total-SCIN-score represent ordered categorical variables. Associations between genotypes and these scales were studied using Chi-square tests and ordinal logistic regression analyses (OLR), the latter approach allowing adjustment for age, and chemotherapy parameters. The risk of an increment within these scales attributed to specific parameters was expressed as odds ratio (OR) with 95% confidence interval (CI). Continuous variables were not normally distributed and are described with median and range. Each of the response variables was analyzed once such that correction for multiple testing was not required. P-values < 0.05 were considered significant, P-values ≥ 0.05 and < 0.10 were reported as of marginal statistical significance. All tests were two-tailed and the analyses were performed using SPSS program version 12.0.2.

### Ethics

The Committee for Medical Ethics of Health Region II of Norway approved the protocol of the study. All patients provided informed consent to participate in the study.

## Results

A total of 238 TCS were eligible for the present study (median age at diagnosis 29 years, range 15–64 years, table 1). The median cumulative cisplatin dose was 397 mg/m^2 ^(range: 81–1571 mg/m^2^). The majority (82%) of patients received not more than one regimen of chemotherapy, which in 87% of such cases comprised BEP (44%) or CVB (44%) and consisted usually of three to four cycles (table 2). The SCIN was completed at median 12 years (range: 4–19 years) after initial diagnosis.

**Table 1:** Demographics of the 238 cisplatin-treated TCSs

Age at diagnosis, years Median (range)	29.3 (14.6 – 63.6)
Observation time, months Median (range)	11.8 (4.3 – 19.3)
Age at survey, years Median (range)	42.3 (22.7 – 73.4)
Histology, number (%)	Seminoma: 44 (18.5%) Non-seminoma: 194 (81.5%)
Stage according to Royal Marsden Hospital, number of patients (%)	I: 72 (30.2%, incl. 2 IM)
	II: 101 (42.4%)
	III: 13 (5.5%)
	IV: 52 (21.8%)
Number of cycles of chemotherapy	1–3: 52 (21.8%) 4: 141 (59.2%) 5–7: 34 (14.3%) >8: 11 (4.6%)
First Regimen (n = 238) Completed by 100% of TCSs	BEP: 104 (43.7%), CVB: 104 (43.7%), BOPVIP: 10 (4.2%), Others: 20 (8.4%)
Second Regimen (n = 44) Completed by 18.5% of TCSs	BEP: 17 (39%), VIP: 12 (27%), EP: 5 (11%), Others: 10 (23%)
Third regimen (n = 8)	VIP: 2, EP: 2, Others: 4
Fourth Regimen (n = 1)	EP: 1
Number of TCSs exposed to the most common cytotoxic agents and cumulative doses among those [mg/m2], median (range)	Cisplatin (n = 238) : 397 (81 – 1571) Bleomycin (n = 226) : 145 (29–212) Vinblastine (n = 105) : 35 (10–51) Etoposide (n = 146) : 1434 (41–4934)

**Table 2:** Association of SCIN-total-score with GST genotypes, age, and chemotherapy parameters by Ordinal Logistic Regression analysis

**Categorical variables**			**Symptoms class of SCIN-total-score**			**Univariate**		**Multiple**†	
**GST genotype**		**Number (%)**	**No (0–5)**	**Moderate (6–9)**	**Severe (10–18)**	**P**	**OR (95% CI)**	**P**	**OR (95% CI)**

P1	**A/A**	99 (41.6)	60 (60.6)	23 (23.2)	16 (16.2)	0.098	2.03 (0.88–4.67)	**0.012**	**3.84 (1.34–10.75)**
	**A/G**	102 (42.9)	53 (52.0)	29 (28.4)	20 (19.6)	0.014	2.83 (1.24–6.46)	**0.003**	**4.95 (1.70–14.45)**
	**G/G**	37 (15.5)	27 (73.0)	10 (27.0)	0 (0)	Reference for *GSTP1-AA *and *-AG*			
T1	**+**	201 (84.5)	118 (58.7)	51 (25.4)	32 (15.9)	0.770	1.11 (0.55–2.22)	0.714	1.17 (0.50–2.73)
	**- (ref.)**	37 (15.5)	22 (59.5)	11 (29.7)	4 (10.8)				
M1	+	134 (56.3)	79 (59.0)	35 (26.1)	20 (14.9)	0.947	0.98 (0.59–1.63)	0.166	1.56 (0.83–2.94)
	**- (ref.)**	104 (43.7)	61 (58.7)	27 (26.0)	16 (15.4)				

**Continuous variables**		**TCSs N (%)**	**Median**	**Minimum**	**Maximum**				

Age at survey (OR for 10 years)		238 (100%)	42.3	22.7	73.4	0.008	1.40 (1.09–1.80)	**0.002**	**1.70 (1.22–2.36)**
OR*	Cisplatin	238 (100%)	397	81	1571	<0.001	1.35 (1.11–1.49)	**0.014**	**1.35 (1.06–1.71)**
	Bleomycin	226 (95.0%)	142	0	212	0.040	1.65 (1.00–2.72)	*0.078*	*1.43 (0.93–2.13)*
	Vinblastine	133 (55.9%)	9.9	0	51	0.535	1.49 (0.41–6.05)	0.319	0.23 (0.01–4.14)
	Etoposide	92 (38.7%)	727	0	4934	0.092	1.00 (1.00–1.00)	0.918	1.00 (0.90–1.11)

*cumulative dose steps: bleomycin (100.000 IE/m^2^), cisplatin (100 mg/m^2^).

† Significant associations (**bold**), marginal significance (*italics*)

In univariate ORL analysis, cumulative doses of cisplatin and bleomycin as well as age at survey were significantly associated with the three-categorical SCIN-total-score (table 2). In multivariate analysis only age at survey and cumulative dose of cisplatin remained significant. Furthermore, polymorphisms in *GSTP1 *had considerable impact: TCSs with *GSTP1-AA *or *GSTP1-AG *had a more than three-fold risk of a more severe symptom-class than those with *GSTP1-GG*. Neither presence of functional GSTT1 nor of functional GSTM1 was significantly associated with the SCIN-total-score.

The cumulative dose of cisplatin was significantly associated with paresthesias in the toes, Raynaud-like phenomena in the toes and with both tinnitus and hearing impairment (table 3). The risk of a more severe symptom-class increased by roughly 1.3 for each step of 100 mg/m^2 ^cumulative cisplatin, the dose corresponding to one cycle of chemotherapy. Age at survey was significantly associated with the severity of each item except from tinnitus. Paresthesias in fingers/toes as well as tinnitus and hearing impairment were significantly associated with polymorphic alleles of *GSTP1 *and/or *GSTM1*. Presence of functional *GSTT1 *was not associated with any of the item-scores. Functional *GSTM1 *increased the risk of hearing impairment by 1.8 (table 3). Furthermore, paresthesias in both fingers and toes as well as Raynaud-like phenomena in the toes showed a trend towards such an association.

**Table 3:** Association of SCIN items and relevant variables by ordinal Logistic Regression analysis

	Age (OR for decadal steps)		Cisplatin (OR for steps of 100 mg/m^2^)		*GSTM1 *(+ vs.- as Reference)		*GSTT1 *(+ vs.- as Reference)		*GSTP1 *(GG vs. AA/AG as Reference)	
	P	OR (95%CI)*	P	OR (95% CI)*	P	OR (95% CI)*	P	OR	P	OR (95% CI)*

Paresthesias fingers	**0.046**	**1.29 (1.01–1.67)**	0.178	1.11 (0.95–1.29)	*0.057*	*1.68 (0.98–2.76)*	0.926	1.03	**0.039**	**0.46 (0.22–0.96)**
Paresthesias toes	**<0.001**	**1.82 (1.40–2.37)**	**<0.001**	**1.34 (1.14–1.57)**	*0.050*	*1.68 (1.00–2.81)*	0.704	0.88	**0.023**	**0.42 (0.20–0.88)**
Raynaud's P. fingers	*0.058*	*1.27 (0.99–1.62)*	**0.021**	**1.27 (1.03–1.39)**	0.235	1.35	0.944	0.98	0.920	0.97
Raynaud's P. toes	**0.022**	**1.34 (1.04–1.72)**	**<0.001**	**1.33 (1.14–1.56)**	*0.072*	*1.60 (0.96–2.66)*	0.355	0.73	*0.052*	*0.49 (0.24–1.01)*
Tinnitus	0.153	1.20	**0.001**	**1.32 (1.12–1.55)**	0.962	1.01	0.107	1.83	**0.008**	**0.33 (0.14–0.74)**
Hearing Impairment	**<0.001**	**1.61 (1.24–2.09)**	**<0.001**	**1.34 (1.14–1.56)**	0.025	1.81 (1.08–3.03)	0.595	1.20	0.553	0.81

*(95% CI) only listed when the association was statistically significant (**bold**) or marginally significant (*italics*)

Presence of both *GSTP1-G *alleles reduced the risk of peripheral paresthesias in the fingers and in the toes, and of tinnitus by at least the factor two. The association between Raynaud-like phenomena in the toes was of a similar magnitude, but only of marginal significance. No symptom was significantly different between TCSs with *GSTP1-AG *and *GSTP1-AA *(data not shown).

**Figure 2:**
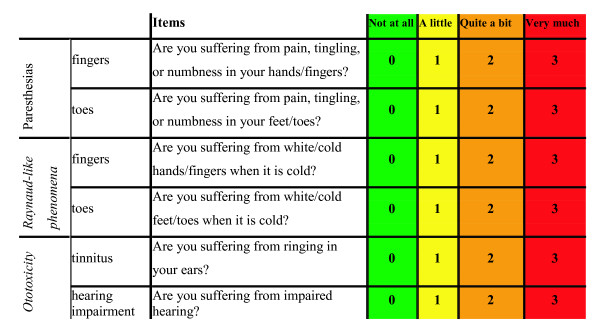
SCIN and its six items with the four possible scores.

In order to illustrate these relations we depicted the scorings of TCSs with either one or none *GSTP1-G *alleles (n = 201) opposed to those with both alleles (n = 37), figure 2. Statistical analysis by χ2 tests for trends revealed significantly protective effect of *GSTP1*-*G *homozygosity against paresthesias in the fingers and toes (p = 0.040 and p = 0.025, respectively), Raynaud-like phenomena in the toes (p = 0.032), and tinnitus (p = 0.003).

## Discussion

After cisplatin-based chemotherapy, the risk of self-reported peripheral paresthesias, Raynaud-like phenomena in the toes, and tinnitus is halved in TCSs homozygous for *GSTP1-G *compared to those with GSTP1-AA/AG. Furthermore, presence of functional *GSTM1 *nearly doubled the risk of hearing impairment.

*GSTP1-GG *proved highly protective against chemotherapy-induced tinnitus (OR = 0.33). Cisplatin leads to loss of outer hair cells in the cochlea [28], and thereby also to hearing impairment [4], which was limited by absence of *GSTM1*. In the subgroup of 173 TCSs in whom audiometry was performed such a beneficial effect of non-functional *GSTM1 *was found for objectively measured cisplatin-induced hearing impairment, which in addition was strongly associated with the genotype *GSTP1-GG *[22]. However, in the present study *GSTP1-GG *did not protect against self-reported hearing impairment. One possible explanation for this lack of association might be a higher sensitivity of audiometric determination of hearing thresholds at 4000 Hz – opposed to self-reported hearing impairment. The cisplatin-induced hearing impairment affects preferentially high frequencies which are no prerequisite for language communication and might thus, despite a measurable deterioration, not be noticed by the affected individuals. Tinnitus appears to be a more sensitive symptom for subjectively experienced cisplatin-induced ototoxicity and its prevalence usually increases with the individual's age [29]. In our TCSs however, the cisplatin-dose is of overriding importance causing *GSTP1-GG *to disclose its protective potential.

Paresthesias may represent a sequel to several drugs applied in our TCSs: Oncovin, vinblastine, and etoposide [30]. Cisplatin-induced paresthesias are experienced by length-dependent symmetrical stocking distribution of sensory symptoms [30]. The protective effect of *GSTP1-GG *presence most likely mirrors an increased tolerance to this drug.

Induction of acute myeloid leukemia is according to Richiardi et al. 38 times more frequent in non-seminoma patients who were treated by etoposide containing chemotherapy compared to the sex-, age-, period, and population-specific incidence rates [31]. Intriguingly, Allan et al. demonstrated a two-fold prevalence of chemotherapy-induced leukemia among cancer survivors with *GSTP1-GG *compared to those with *GSTP1-AA *or *GSTP1-AG *[32]. This risk increased to the odds ratio of four in patients who had received known GSTP1 substrates like e.g. cyclofosfamide, etoposide, or adriamycin.

Induction of leukemia and neurotoxicity by chemotherapy is apparently curbed by strictly opposite GSTP1 polymorphisms. This seemingly contradiction might be due to substrate specificity: Experiments with E.coli cloned with human polymorphic *GSTP1 *indicate that topoisomerase inhibitors are preferentially detoxified by *GSTP1-A *whereas cisplatin is best inactivated by *GSTP1-G *[33]. The examined SNP A→G causes substitution of isoleucine by valine in GSTP1 at codon 105, a residue regulating the affinity and detoxification efficacy for electrophilic substrates [34]. E.coli with human *GSTP1-G *have a doubled cytoprotection against cisplatin as compared to those with *GSTP1-A*. Recently, *GSTP1-G *was demonstrated to protect patients with gastric cancer against oxaliplatin-induced neuropathy [35]. Taken together, studies in both bacteriae and cancer patients indicate that *GSTP1-GG *protects its carriers against platinum-induced toxicities.

The observed beneficial absence of GSTM1 could be explained by a competition on glutathione (GSH) as substrate of both GSTM1 and GSTP1. Both GST-M1 and GST-P1 are co-expressed in the mammalian cochlea and in dorsal root ganglion whose cells are linked to cisplatin-induced paresthesias [17,18,36]. The latter may detoxify cisplatin more effectively and might unfold more of its protective potential when the competing GSTM1 is not present. Supplementation of GSH demonstrated in a small randomized placebo-controlled study protection against oxaliplatin-induced neurotoxicity [37].

Alteration of intracellular apoptosis pathways might represent an alternative or additional explanation to varying detoxification efficacies: GSTP1 monomers bind- and thus inactivate the stress-inducible Jun N-terminal kinase (JNK) [38]. Oxidative stress releases GSTP1 from JNK, which in turn activates the expression of GSTP1 and other genes involved in apoptosis and cytoprotection [39]. Inhibition of JNK in cisplatin-treated guinea pigs increased ototoxicity [40]. Hypothetically, protection against cisplatin-induced toxicities might be due to less effective JNK inactivation by the *GSTP1-G-*derived enzyme GSTP1^105^Val. Intriguingly, GSTM1 was found to be a binding partner of the apoptosis signal-regulating kinase 1 (ASK-1). Binding of ASK-1 by GST-M1 inhibits its function and prevents thereby activation of JNK and p38 pathways [41]. Deletion of GST-M1, resulting in absence of this functional enzyme might thereby increase the risk of cisplatin-induced apoptosis. We admit that both presented functional explanations of our findings, i.e. interaction with cell signaling factors and competition on glutathione as substrate for both GST-P1 and GST-M1, remain theoretical ones. However, these hypotheses appear readily refuted or supported by cell culture experiments.

The role of antioxidants for neuro-protection in chemotherapy receiving patients is debated. Pace et al. demonstrated Vitamin E to prevent cisplatin-induced neurotoxicity to some extent,[42] however, a trial examining calcium- and magnesium salts in oxaliplatin receiving cancer patients had to be stopped due to a lower response rate compared to placebo[43]. It might be worthwhile to examine neuro-protective effects of antioxidants with respect to potentially important polymorphisms. Glutathione's protective potential might correlate with GST polymorphisms. Hopefully, translation of these findings into clinical practice includes prospective assessment of neuro-protective agents and their modulation by functional polymorphisms.

Prediction of anticipated toxicities might help patients and oncologists to choose individualized treatment. Therefore, *GST *genotyping might become a helpful tool for avoidance of chemotherapy-induced long-term toxicities. The strongest limitation of our study might represent the reliance on self-reported symptoms only. Objectively measured findings, e.g. nerve conduction velocity and Doppler-flow examination of the digital arteries after cold exposure, would have facilitated the distinction between paresthesias and Raynaud-like phenomena. Furthermore, pre-treatment evaluations are not available, precluding assessment of intra-individual treatment-related changes over time. However, the high compliance-rate of this survey (81%) conducted more than ten years after treatment is considered quite unique and the size and homogeneity of the sample of TCSs might partly compensate for the above mentioned limitations. The observed strong association between SCIN and the cumulative cisplatin-dose permits evaluation of protective genotypes.

In conclusion, presence of both *GSTP1-G *alleles and/or absence of functional GSTM1 protect its carriers against several chemotherapy-induced long-term toxicities. Before clinical consequences can be discussed, these results should be corroborated in an independent sample of cancer patients. However, the concordance between audiometrically evaluated cisplatin-induced hearing impairment and the observations presented here builds a strong case for the presence of relevant associations between GST polymorphisms and long-term chemotherapy-induced toxicities.
